# Precision and Progress: Machine Learning Advancements in Plastic Surgery

**DOI:** 10.7759/cureus.41952

**Published:** 2023-07-16

**Authors:** Mohd Altaf Mir, Rajesh Maurya

**Affiliations:** 1 Burns and Plastic Surgery, All India Institute of Medical Sciences, Bathinda, Punjab, IND

**Keywords:** scar appearance, outcome analysis, pre-surgical planning, robotic surgical procedures, machine leaning

## Abstract

Machine learning has emerged as a powerful tool in various healthcare domains, and its application in plastic surgery has shown significant promise. Plastic surgery aims to enhance and reconstruct physical appearance and function, making it an ideal field for integrating machine learning techniques. This abstract presents an overview of the applications, challenges, and potential benefits of machine learning in plastic surgery. One of the key areas where machine learning has been applied is in the preoperative assessment and surgical planning process. By analyzing large datasets of patient images and clinical data, machine learning algorithms can assist plastic surgeons in predicting surgical outcomes, identifying optimal surgical techniques, and minimizing potential complications. These algorithms can learn from past cases and provide valuable insights to improve surgical decision-making and optimize patient care. Furthermore, machine learning has shown promise in facial recognition and analysis, which is crucial in plastic surgery procedures involving the face. Algorithms can accurately detect facial landmarks, assess facial symmetry, and simulate potential surgical outcomes. This technology gives plastic surgeons a more comprehensive understanding of the patient's facial structure and aids in designing personalized treatment plans. Additionally, machine learning algorithms have been employed to automate the analysis of large-scale clinical databases, assisting in identifying patterns, risk factors, and treatment outcomes. By leveraging these algorithms, plastic surgeons can gain valuable insights into patient populations, surgical trends, and postoperative complications. This information can inform clinical decision-making, improve patient safety, and enhance the overall quality of care. Despite the numerous advantages, several challenges need to be addressed when integrating machine learning into plastic surgery. These include the need for high-quality and diverse datasets, algorithm interpretability, ethical considerations, and regulatory compliance.

## Editorial

Machine learning is significantly advancing in various fields, including healthcare and medicine. Plastic surgery, a specialized medical branch, has also seen the integration of machine learning techniques for various purposes. Below are some areas where machine learning is being used in plastic surgery [1-3]. Machine learning algorithms can analyze facial features and patterns to aid in facial recognition and analysis. This technology can be utilized in plastic surgery for preoperative planning, simulation, and prediction of postoperative outcomes. Using machine learning algorithms, surgeons can assess the impact of different surgical procedures on a patient's facial appearance and make informed decisions [1,2]. Machine learning algorithms can be employed to process and analyze medical images, such as X-rays, CT scans, and MRI scans. In plastic surgery, these algorithms can assist in identifying specific anatomical structures, evaluating tissue characteristics, and detecting abnormalities or tumors. This aids surgeons in preoperative assessment and surgical planning [3,4]. This also allows for more precise and personalized surgical planning [1-5]. Machine learning algorithms can automatically segment different tissues in medical images. In plastic surgery, this can be useful for accurately delineating anatomical structures like skin, muscles, and bones. Automated tissue segmentation can save time and improve the accuracy of surgical planning [1-5]. By leveraging machine learning techniques, surgeons can analyze large patient records and outcomes datasets to develop predictive models. These models can help estimate the potential risks and complications associated with specific plastic surgery procedures. Surgeons can use these predictive models to counsel patients, set realistic expectations, and devise personalized treatment plans [1-5]. It can be integrated into surgical navigation systems to provide real-time guidance during procedures. By analyzing intraoperative data, such as images and sensor readings, these algorithms can help surgeons identify critical structures, optimize incisions, and ensure accurate implant placement [1-5]. Machine learning algorithms can be combined with augmented reality (AR) and virtual reality (VR) technologies to create immersive surgical planning and training experiences. Surgeons can visualize and manipulate virtual 3D models of patients' anatomy, enabling a better understanding of complex anatomical structures and practicing surgical techniques in a simulated environment [1-5]. Robotic-Machine learning algorithms can be integrated into robotic systems used in plastic surgery. These algorithms enable precise control and enhance the capabilities of robotic surgical platforms. Surgeons can benefit from improved dexterity, stability, and accuracy during procedures, leading to better patient outcomes [1-5]. It can monitor and analyze postoperative data to detect early signs of complications. By analyzing patient-reported outcomes, vital signs, and other relevant data, algorithms can identify deviations from normal recovery patterns and alert healthcare providers to potential complications, enabling prompt intervention and improved patient care [4]. Machine learning algorithms can be trained to assess the severity of scars and predict their evolution over time. By analyzing various features of scars, such as color, texture, and thickness, these algorithms can provide objective measurements and recommendations for scar management, including treatment options and monitoring progress [3,4]. It can assist in patient selection for specific plastic surgery procedures. By analyzing patient data, such as medical history, demographics, and imaging, algorithms can identify individuals who are likely to achieve optimal outcomes from a particular procedure. This personalized approach helps improve patient satisfaction and reduce the risk of adverse effects [1-5]. Machine learning algorithms can process and analyze large amounts of clinical and research data to identify trends, patterns, and associations. Plastic surgery researchers can leverage these algorithms to discover new insights, refine surgical techniques, and improve the overall quality of care [3]. It is important to note that while machine learning has shown promise in plastic surgery, it should always be used in conjunction with the expertise and clinical judgment of experienced surgeons. These technologies serve as tools to assist and augment surgical decision-making rather than replacing the skills and knowledge of plastic surgeons.
